# Nasal Carriage and Antimicrobial Susceptibility of *Staphylococcus aureus *in healthy preschool children in Ujjain, India

**DOI:** 10.1186/1471-2431-10-100

**Published:** 2010-12-29

**Authors:** Ashish Pathak, Yogyata Marothi, Rama V Iyer, Binita Singh, Megha Sharma, Bo Eriksson, Ragini Macaden, Cecilia Stålsby Lundborg

**Affiliations:** 1 Division of Global Health (IHCAR), Department of Public Health Sciences, Nobels väg 9, Karolinska Institutet, Stockholm, 17177, Sweden; 2 Department of Pediatrics, R.D. Gardi Medical College, 456010, Ujjain, India; 3 Department of Microbiology, R.D. Gardi Medical College, 456010, Ujjain, India; 4 Department of Pharmacology, R.D. Gardi Medical College, 456010, Ujjain, India; 5 Nordic School of Public Health, Göteborg, Sweden; 6 St Johns Research Institute, Bangalore, India

## Abstract

**Background:**

There is increasing evidence that community acquired *S. aureus *infections are spreading among healthy children. Nasal colonization with *S. aureus *plays pivotal role in the increasing prevalence of resistant community acquired *S. aureus *infections worldwide. A regular surveillance system is important in ensuring quality of patient care. The aim of the study was to assess the prevalence of and the factors associated with nasal carriage of *S. aureus *and its antibiotic sensitivity pattern among healthy children in Ujjain, India.

**Methods:**

A prospective study was done in paediatric outpatient clinics of R.D. Gardi medical college Ujjain, India. Healthy children from 1 month to 59 months of age were included. Information on previously known risk factors for nasal colonization was collected using a pre-tested questionnaire. Swabs from anterior nares were collected and transported in Amies transport media with charcoal and cultured on 5% sheep blood agar. Antibiotic sensitivity tests were performed using Kirby Bauer's disc diffusion method according to performance standards of Clinical and Laboratory Standard Institute guidelines.

**Results:**

Of the 1,562 children from 1-month up-to five years of age included in the study 98 children tested positive for nasal carriage of *S. aureus*. The prevalence of nasal carriage of *S. aureus *was 6.3% (95% CI 5.1-7.5) out of which 16.3% (95% CI 8.9-23.8) were methicillin-resistant *S. aureus *(MRSA). The factors associated with nasal carriage were "child attending preschool" (OR 4.26, 95% CI 2.25-8.03; *P *= 0.007) or "school" (OR 3.02, 95% CI 1.27-7.18; *P *< 0.001) and "family size more than 10 members" (OR 2.76 95% CI 1.06-7.15; *P *= 0.03). The sensitivity pattern of isolated *S. aureus *showed resistance to commonly used oral antibiotics while resistance to glycopeptides was not noted.

**Conclusions:**

We found a relatively low rate of nasal carriage of *S. aureus *in children below five years when compared to children of older age groups in India. Yet, prevalence of MRSA was relatively high.

## Background

*Staphylococcus aureus *is a common pathogen responsible for community as well as hospital-associated infections. The infections caused by *S. aureus *have clinical range from minor skin infections to severe life threatening infections [1]. There is increasing evidence that community acquired methicillin-resistant *S. aureus *(CA-MRSA) is spreading among healthy individuals, especially children [2].

The anterior nares have been shown to be the main reservoir of *S. aureus *in both adults and children. The *S. aureus *is transmitted to nares by contaminated hands and from surfaces where it can survive for months [3]. Nasal carriage of *S. aureus *acts as endogenous reservoir for clinical infections in the colonized individual but also as a source of cross-colonization for community spread. The spread of colonization occur especially in close contact areas like schools, pre-schools or households [2] probably by the contaminated hands and surfaces.

The individuals colonized with *S. aureus *(both CA-MRSA and CA-MSSA ie, community acquired methicillin-sensitive *S. aureus*) tend to have a complicated clinical course from a disease originating from their endogenous *S. aureus *[3]. The complicated clinical course results from increasing resistance in *S. aureus *isolates and also because the bacteria can cause deep-seated infections and sepsis [3,4]. The difficulties in treating these deep-seated infections and sepsis require urgent measures to prevent further spread of MRSA. Setting up a bacterial surveillance system is one of the strategies to understand the epidemiology of MRSA to guide local antibiotic policy and to compare resistance patterns with other international surveillance systems. An improved understanding of epidemiology and resistance mechanisms of community acquired MRSA is required for designing better preventive strategies for further spread of resistance.

In India studies have been done in adults in intensive care units [5-8] and among patients at high risk of *S. aureus *infection [9] but studies on prevalence of nasal carriage and antibiotic susceptibility pattern of *S. aureus *in Indian children are few [10,11]. Further, prevalence studies in India have been done in children aged between 5 to 15 years of age but little is known about epidemiology of *S. aureus *in children below five years of age. Ramana *et al *[10] studied 392 children, aged 5 to 15 years and showed a carriage rate of 16% for *S. aureus *of which 19% were MRSA. Other Indian study by Chatterjee *et al *[11] using polymerase chain reaction (PCR) studied 489 children, aged 5 to 15 years and showed a prevalence of 52.5% for *S. aureus *of which 3.8% were MRSA.

The present study is done to understand the epidemiology of nasal carriage of *S. aureus *in view of paucity of studies in healthy children below five years of age in India. The first objective of the study was to estimate the prevalence of nasal carriage of *S. aureus *and MRSA as well as to identify factors associated with such carriage among healthy children (below 5 years of age). The second objective was to study the sensitivity patterns of the isolates.

## Methods

This was a prospective study conducted during 15 months from November 2007 to February 2009.

### Study setting

The study was conducted in paediatric outpatient clinics of two hospitals, the V.D. Gardi Charitable Trust Hospital (UCTH; the non-teaching hospital) and the C. R. Gardi Hospital (CRGH; the teaching hospital). UCTH is a 350 bedded hospital located in the city of Ujjain, whereas the 570 bedded CRGH is situated in a semi-urban area approximately six km from Ujjain city. C.R. Gardi Hospital is attached to R.D. Gardi Medical College (RDGMC). Both the hospitals cater to the population of Ujjain district, which has a population of 1,710,000 inhabitants with a population density of 281 per sq km [12]. Most hospital visits in both the hospitals are unscheduled visits from surrounding rural areas where agriculture is the main source of income.

### Study participants

In the paediatric outpatients, healthy children from 1 month up-to 59 months of age visiting for routine immunization were included to identify nasal carriage of *S. aureus*. Children with any suspected infection including upper respiratory tract infection and those requiring admission or emergency care were not included. Also, patients with current skin infections were not included.

Parent or caregiver accompanying the child for immunization was interviewed by one of the study assistants and a questionnaire was filled-in. The questionnaire contained patient demographic characteristics and information on suspected factors associated with nasal carriage of *S. aureus *(for details see Table 1). A pilot study was done for one month (May 2007) each in both the hospitals using the questionnaire to test logistics and train the study assistants.

**Table 1:** Multiple logistic regression analysis† of factors associated with Staphylococcus aureus nasal carriage in children between 1 to 59 months of age in Ujjain

		*Staphylococcus aureus*				
Factor	Totals (% within column)	Positive No. (%)	Negative No. (%)	*P*	Adjusted OR	95% CI
Sex						
Boy	902 (57.8)	60 (6.7)	842 (93.3)	0.67	0.90	0.59-1.39
Girl	660 (42.2)	38 (5.8)	622 (94.2)			
Age group*						
1-6 months	614 (39.3)	41 (6.7)	573 (93.3)	1	1	-
7-12 months	218 (13.9)	9 (4.1)	209 (95.9)	0.20	0.58	0.26-1.29
13-24 months	248 (15.8)	12 (4.9)	236 (95.1)	0.16	0.56	0.25-1.26
25-59 months	482 (31.0)	36 (7.5)	446 (92.5)	0.12	0.48	0.18-1.23
Current breastfeeding status						
Yes	812 (52.0)	51 (6.4)	761 (93.6)	0.72	1.08	0.58-2.00
No	750 (48.0)	47 (6.3)	703 (93.7)			
Child attends*						
No school	1115 (71.4)	1062 (95.2)	53 (4.8)	1	1	-
Preschool	143 (9.2)	123 (86)	20 (14.0)	< 0.001	4.26	2.25-8.03
School	304 (19.4)	279 (91.8)	25 (8.2)	0.012	3.02	1.27-7.18
Family size*						
Less than or equal to 4	449 (28.7)	21 (4.7)	428 (95.3)	1	1	-
Between 5-10	1061 (68.0)	70(6.7)	991 (93.3)	0.12	1.50	0.89-2.54
More than 10	52 (3.3)	7 (13.5)	45 (86.5)	0.03	2.76	1.06-7.15
Education of the mother*						
Illiterate	593 (38.0)	26 (4.4)	567 (95.6)	1	1	-
Up-to primary	537 (34.2)	44 (8.2)	491 (91.8)	0.10	1.65	0.90-3.03
Up-to higher secondary	255 (16.3)	20 (8.2)	235 (91.8)	0.24	1.51	0.74-3.07
Graduate or post graduate	179 (11.5)	8 (4.5)	171 (95.3)	0.95	1.02	0.42-2.51
Occupation						
Self employed	990 (63.4)	52 (5.2)	938 (94.8)	0.33	0.77	0.45-1.29
Salaried	572 (36.6)	47 (8.2)	525 (91.8)			
Antibiotic usage in last 2 weeks						
Yes	330 (26.8)	25 (7.6)	305 (92.4)	0.92	0.97	0.60-1.57
No	1232 (73.2)	74 (6)	1158 (94)			
Hospitalization in the last 2 weeks						
Yes	137 (8.8)	17 (12.4)	120 (87.6)	0.08	1.79	0.92-3.47
No	1425 (91.2)	82 (5.8)	1343 (94.2)			
Hospital visit in the last 2 weeks						
Yes	370 (23.7)	31 (8.4)	339 (91.6)	0.25	1.32	0.81-2.14
No	1192 (76.3)	68 (5.7)	339 (94.3)			

* Logistic regression was used with first value within variable acting as reference value

^†^A complete case analysis with n = 1562 was used. Variables entered: sex (boys versus girls), age group (13 months to 24 months and 13 to 24 months versus 1-12 months), current breastfeeding status (yes versus no), child attends no school, preschool, school (no school/preschool versus preschool and school), family size (between 5 to 10 members and more than 10 members to less than or equal to 4 members), education of the mother (up-to primary, up-to higher secondary and graduate or postgraduate versus illiterate), occupation of the breadwinner (self employed versus salaried), antibiotic use in the last 2 weeks (no versus yes), hospitalization in the last 2 weeks (no versus yes), hospital visit in the last 2 weeks (no versus yes).

Verbal consent was obtained from all caregivers after explaining the purpose of the study. The ethics committee of R.D. Gardi Medical College approved the study (approval number 41/2007).

### Collection of nasal samples and laboratory methods

Sampling for each participating child was performed by twice rotating a sterile cotton swab, pre-wetted with sterile saline in the vestibule of both anterior nares. The collected samples were transported in Amies transport media with charcoal (HiMedia, Mumbai, India) at a temperature between 4-8°C to the microbiology laboratory at R.D. Gardi Medical College, within 4 hours of collection.

Swabs were cultured on 5% sheep blood agar, incubated at 35 ± 1°C and examined for growth after 24-48 hours. Colonies growing on blood agar were identified as *S. aureus *by their typical colony morphology, Gram's staining, anaerobic utilization of glucose and mannitol, catalase production and tube coagulase test. Screening for methicillin resistance was done using cefoxitin disk screen test and 6 μg/ml of oxacillin in Mueller-Hinton agar supplemented with NaCl (4% w/v; 0.68 mol/L) according to Clinical and Laboratory Standard Institute (CLSI) guidelines [13].

Antibiotic sensitivity tests were performed using Kirby Bauer's disc diffusion method according to performance standards of CLSI [13]. *S. aureus *ATCC 25923 was used as control strain. The panel of antibiotics tested included those that are recommended by CLSI or are commonly used locally in empirical treatment of *S. aureus *infections. The panel evolved after discussions with paediatricians and clinical microbiologists within and outside the research group. Susceptibility testing was done and results are presented for the following most important antibiotics: co-trimoxazole, ampicillin, co-amoxiclav, ciprofloxacin, levofloxacin, ceftriaxone, erythromycin, clindamycin, doxycycline, chloramphenicol, tetracycline, gentamicin, amikacin, linezolid, teicoplanin and vancomycin. Inducible clindamycin resistance was detected by double disk diffusion test (D test); performed by placing the clindamycin and erythromycin disks 15 mm apart [14]. For both MSSA and MRSA we defined multi-drug resistant (MDR) isolates as those resistant to 3 different antibiotics ie, co-trimoxazole, ciprofloxacin and erythromycin.

### Statistical analysis

The data was entered in EpiData Entry (version 3.1) and then transferred to Stata 10.0 (Stata Corp. College Station, Texas, USA) software for statistical analysis. Prevalence of *S.aureus *and MRSA were estimated with 95% confidence intervals. The relationship between each variable and the outcome (nasal carriage of *S. aureus*) was explored using odds ratios (OR). Crude OR's were calculated from two by two tables. A given variable was entered in the final multiple logistic model if the bivariate analysis yielded a P value less than 0.1. All the variables were adjusted for age and sex. A complete case series analysis was used. The independent variables included were: sex (boys versus girls), age group (7 to 12 months, 13 to 24 months and 25 to 59 months versus 1 to 6 months), current breastfeeding status (yes versus no), child attends no school, preschool, school (no school/no preschool versus preschool, school), family size (between 5 to 10 members and more than 10 members to less than or equal to 4 members), education of the mother (up-to primary, up-to higher secondary and graduate or postgraduate versus illiterate), occupation of the breadwinner (self employed versus salaried), antibiotic use in the last 2 weeks (yes versus no), hospitalization in the last 2 weeks (yes versus no), hospital visit in the last 2 weeks (yes versus no). Chi-square tests were used to test for statistical significance (5%).

## Results

### Study population

Of the 1600 caregivers approached 1,562 (98%) consented for their child to participate in the study; 902 were boys (58%) and 660 girls (42%). Almost equal numbers of children were enrolled from the two hospitals. Thirty-nine percent of the children were between 1 and 6 months of age, 31% were between 25 and 59 months.

The prevalence of breastfeeding before 6 months of age was 92%; however whether breastfeeding was exclusive or not was not documented. About 50% of the 482 children above 3 years of age went to school or preschool and 9% to 17% of children in the different age categories utilized *anganwadi *which are Government of India run mother and child care services (pre-school) intended for children between 0 to 6 years of age.

Thirty-eight percent of mothers were uneducated or educated with only primary education. Most of the mothers (84%) with graduate or postgraduate degree were enrolled from the non-teaching whereas 64% of all illiterate mothers were enrolled from the teaching hospital.

Antibiotic usage in the last 2 weeks was reported for 26% of children. A history of hospital visit other than for immunization in the last 2 weeks was noted for 27% of all children and 8.7% had a history of hospitalization in the last 2 weeks (Table 1).

### Factors associated with nasal carriage

The prevalence of nasal carriage of *S. aureus *was 6.3% (95% CI 5.07-7.47). Out of 98 *S. aureus *isolates 16.3% (95% CI 8.87-23.77) were MRSA. The factors associated with colonization with *S. aureus *are shown in Table 1. The statistically significant OR observed in the multiple logistic regression models with nasal carriage of *S. aureus *as dependent variable were "child attending preschool" (OR 4.26, 95% CI 2.25-8.03; *P *= 0.007) or "school" (OR 3.02, 95% CI 1.27-7.18; *P *< 0.001) and "family size more than 10 members" (OR 2.76 95% CI 1.06-7.15; *P *= 0.03).

### Antibiotic sensitivity pattern

**Figure 1:**
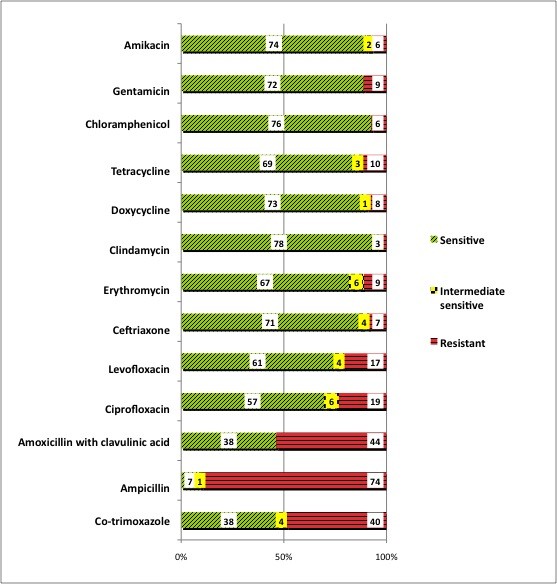
**Antibiotic sensitivity patterns of 82 isolates of Methicillin susceptible *Staphylococcus aureus *(MSSA) in children from 1-59 months of age in Ujjain**.

The in-vitro antibiotic sensitivity pattern of 82 isolates of methicillin susceptible *S. aureus *(MSSA) is shown in Figure 1. Resistance to commonly used oral antibiotics, ampicillin (90%), co-amoxiclav (54%), co-trimoxazole (49%), ciprofloxacin (23%) and erythromycin (11%) was noted in MSSA isolates. Among the erythromycin resistant strains of MSSA 15% were clindamycin inducible. Co-resistance to a combination of amikacin with different classes of antibiotics is as follows: ampicillin with amikacin (6%), co-amoxiclav with amikacin (4%), ciprofloxacin with amikacin (1%) and ceftriazone with amikacin (1%). Co-resistance to ciprofloxacin and erythromycin was (6%).

Four MSSA isolates were MDR (resistant to 3 different antibiotics ie, co-trimoxazole, ciprofloxacin and erythromycin) and two of the carriers of these isolates had reported history of hospitalization in the previous two weeks and both had received ceftriaxone during hospitalization.

No resistance was noted to vancomycin, linezolid or teicoplanin. Higher sensitivity was noted to clindamycin (94%), chloramphenicol (87.5%), doxycycline (82.7%), amikacin (81.3%) and erythromycin (76.6%).

**Figure 2:**
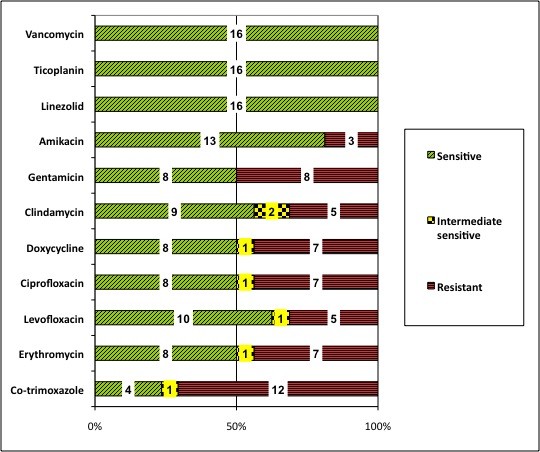
**Antibiotic sensitivity patterns of 16 isolates of Methicillin resistant *Staphylococcus aureus *(MRSA) in children from 1-59 months of age in Ujjain**.

The antibiotic sensitivity pattern of 16 isolates of MRSA is shown in Figure 2. The MRSA isolates showed resistance to co-trimoxazole (75%), doxycycline (44%), ciprofloxacin (44%), levofloxacin (31%) and erythromycin (44%). Among the erythromycin resistant strains of MRSA 35% were clindamycin inducible. Co-resistance to ciprofloxacin and erythromycin (25%), erythromycin and amikacin (19%), doxycycline and amikacin (12.5%) ciprofloxacin and amikacin (12.5%), levofloxacin and amikacin (12.5%) doxycycline and levofloxacin (6%), clindamycin and amikacin (6%), was noticed.

Among the 16 MRSA isolates 3 (19%) were MDR. Among the carriers of these isolates one child was hospitalized in the last two weeks and had received antibiotic co-amoxiclav during the stay.

## Discussion

This is the first study from India reporting *S. aureus *nasal carriage rate in healthy children below 5 years. The study showed that 6.3% of healthy children below 5 years attending outpatient clinics of two hospitals in Ujjain carried *S. aureus *out of which 16.3% were MRSA. Ramana KV *et al *[10] reported a prevalence of 16% for *S. aureus*, 19% of which were MRSA among school going children (5 to 15 years) in Narketpally, Andhra Pradesh, India. However, the factors associated with acquisition of *S. aureus *where not evaluated in that study. In another study of 489 school children (5 to 15 years of age) by Chatterjee SS *et al *[11] using enriched culture media and PCR assay for *mecA *gene, colonization was found in 256 (52.5%) children. Out of these 3.9% were MRSA. They identified living in mud-thatch housing as factor associated with nasal carriage in their study.

The carrier rates of *S. aureus *and MRSA reported in different studies are shown in Table 2. It can be noted that *S. aureus *colonization is generally lower in resource poor countries and varies in the same community over time with increasing rates in the more recent studies. One possible explanation of high nasal carriage of *S. aureus *in resource rich countries could be low rates of exposure to antigens due to better personal hygiene leading to decreased clearing of pathogens in the tested patients [15].

**Table 2:** Carrier rates of S. aureus and MRSA reported in different studies

Authors	Year of publication	Country	Age group included	Study setting	Sample size	*S. aureus *nasal carriage rate	MRSA carriage rate
Ramana KV *et al *[10]	2009	India	5-15 year	Schools	392	16%	19% of the *S. aureus *samples
Chatterjee SS *et al *[11]	2009	India	5-15 year	Schools	489	52.5% (PCR)	3.89%
Ciftci H *et al *[16]	2007	Turkey	4-6 years	Schools	1134	28.4%	0.3%
Ko KS *et al *[28]	2008	Korea	1-11 years	Hospital outpatients	296	32.1	18.9% of the *S. aureus *samples
Lamaro-Cardosa J *et al *[21]	2009	Brazil	2 months to 5 years	Day care centres	1,192	31.1%	1.2%
Lebon A *et al *[22]	2008	Netherlands	1.5 months and 14 months	Community based longitudinal study	443	12.9% at 1.5 months and 12.9% at 14 months	Not reported
Lee GM *et al *[23]	2009	USA (Massachusetts)	3 months to less than 7 years	Community	1,968	14.1 in 2003-4 and 14.6% in 2006-7	0.2% in 2003-4 and 0.9% in 2006-7
Lo WT *et al *[29]	2007	Taiwan	Less than 7 years	Day care centres	68	25%	13.2%
Oguzkaya-Artan M *et al *[30]	2008	Turkey	0-6 years	Primary care centre	200	18%	5.6%
Regev-Yochay G *et al *[20]	2009	Israel	0-40 months	Primary care centre	4,648	7.6%	Low (3 children)

The prevalence of colonization with *S. aureus *has previously been shown to be age dependent [2-4,16-18]. The prevalence varied across different age groups in our study with lower prevalence in the first six months of life although these differences were not statistically significant. The peak of colonization with a respiratory pathogen may be seen at 2-3 years of age [18]. During this age a lot of pathogens compete for colonization of the anterior nares; examples are pneumoccoci, *Haemophilus influenzae*, *Moraxella catarrhalis *and *S. aureus*. Bacterial interference, phenomenon by which colonization by one bacterial strain prevents colonization by another strain, plays an important role in establishing or eliminating one bacterial strain over another [15].

In our study the children attending school or *anganwadi *(Government of India run mother and child (0 to 6 years) services) were shown to have significantly higher prevalence of carriage. The finding is consistent with the fact that large family size with 10 or more members had higher carriage prevalence as compaired to families with less than or equal to 4 members. This might be due to poor hygiene and overcrowding. Recent studies have demonstrated the importance of close contacts within households [19] and with parents [20] in spread of *S. aureus *carriage among children residing in the same household. In the above two studies the children shared the genotype of *S. aureus *with one of the parents, more often the mother indicating the importance of close contact within the family.

Hospitalization in the recent past was not a factor significantly associated with nasal carriage in our study. However, hospitalization has been demonstrated by other studies [3,16,21-23] as a significant risk factor. Similarly, hospital visit is considered a risk factor [3] for acquiring *S. aureus *carriage however; in our study hospital visit was not identified as a statistically significant factor associated with nasal carriage.

In our study we did not find exposure to antibiotics in the past 2 weeks as an important factor associated with *S. aureus *carriage. However, in our study too, two of the four children carrying MDR isolate of MSSA and one of the three children carrying MDR MRSA isolates had reported history of hospitalization. Many of these children had received antibiotics in the last two weeks.

A meta-analysis by Costelloe *et al *[24] discussed the effect of antibiotic exposure on resistance over 12 months in MRSA isolated from skin abrasions. The authors reported a pooled OR of 1.04 with confidence interval crossing the null. But, the meta-analysis had only three studies included in it. Also, as in the present study, data on antibiotic exposure was by parental reports only, which was the case in two of the three studies included in the meta-analysis [24]. Antibiotic use is one of the most important determinants of antibiotic resistance [24]. A high antibiotic use rate is reflected by the fact that 26% of children enrolled in the study had received an antibiotic in the previous two weeks. Antibiotic stewardship programmes that promote judicious use of antibiotic are urgently needed and could prove to be more cost effective then targeted screening based on risk factors, isolation of the carriers and decolonization [24]. Simple hygiene measures like hand washing are effective in preventing spread of resistant organisms in the community. The importance of hygiene is exemplified in an intervention program in Swedish day care centres, which introduced alcohol-based hand washing for children. This intervention significantly reduced (by 12% points) children's absence from the day care [25].

Early in the global epidemic of MRSA there was a clear distinction in susceptibility pattern of CA-MRSA and hospital associated MRSA (HA-MRSA), with CA-MRSA being susceptible to most antibiotics and typically highly sensitive to clindamycin [26]. Recent studies, however, demonstrate that the boundaries between these two entities are getting blurred [21,23,26]. This may be due to simultaneous horizontal transmission of multiple HA-MRSA strains in the community; indicating established transmission of HA-MRSA in the community for some time [27]. The *S. aureus *isolates in our study show high sensitivity to clindamycin but have high resistance to co-amoxiclav. This may represents a scenario of a late stage of spread in the community, albeit at a low rate [26,27].

In MRSA isolates resistance was seen to antibiotics that are important for empirically treating severe infections. These antibiotics include doxycycline (44%), levofloxacin (31%) and clindamycin (5%). Resistance to levofloxacin and clindamycin is a cause of concern because of their therapeutic value in treating serious *S. aureus *infections in high-risk patients. However, it is important to note that the present results are of carriage state and not clinical infections.

Our study has limitations; firstly, we did not use an enriched media for culture of *S. aureus*, which might have reduced our detection rates. Secondly, we did not measure the minimum inhibitory concentration (MIC), which is the recommended method especially for glycopeptide antibiotics. However, we did not notice resistance to gycopeptides in our study. Also, we did not do confirm MRSA status by doing *mecA *gene due to financial constrains. Thirdly, our study is an observational study conducted in hospital setting although with healthy children from the community. A community based cohort design with sampling of a given child at various ages would have identified persistent carriage (carriage over time), which is more important source for community spread.

## Conclusions

This is the first study among Indian children below five years of age studying the prevalence of nasal carriage and showed a prevalence of 6.3% for *S. aureus *16.3% of which were MRSA. The study shows that attending pre-school or school and living in a large family was associated with nasal carriage of *S. aureus*. More studies with cohort design are needed to accurately assess the epidemiology of *S. aureus *nasal carriage in various geographical locations.

## Competing interests

The authors declare that they have no competing interest.

## Authors' contributions

AP, YM, RVI, MS, BE, RM and CSL participated in the conception and design of the study and revising the paper critically for substantial intellectual content. AP was responsible for the data collection, performed the data analysis and drafted the manuscript. YM, RVI and BS were responsible for laboratory testing. BE contributed to the statistical analysis. All authors read and approved the final manuscript.

## Pre-publication history

The pre-publication history for this paper can be accessed here:

http://www.biomedcentral.com/1471-2431/10/100/prepub
